# Common pathways in the neurobiology of post-COVID conditions and of Mood Disorders

**DOI:** 10.1192/j.eurpsy.2025.42

**Published:** 2025-08-26

**Authors:** F. Benedetti

**Affiliations:** 1 University Vita-Salute San Raffaele; 2 Division of Neuroscience, IRCCS Ospedale San Raffaele, Milan, Italy

## Abstract

**Abstract:**

Mood disorders (MD) have been consistently associated with alterations in the immune system. Evidence suggests a condition of systemic low-grade inflammation with abnormally decreased adaptive, increased innate immunity, and with higher levels of circulating cytokines, higher macrophage/monocyte inflammatory activation patterns, and higher neutrophils to lymphocyte counts. A dynamic pattern of premature immunosenescence and partial T cell defect starting early in adolescence, involving a reduction of naïve T cells and an expansion of memory and senescent T cells, parallels lifetime recurrence of illness episodes, worsening outcomes and fostering chronicity. Consistent systematic reviews and meta-analyses affirm that COVID-19 survivors show persistent psychopathology and neurocognitive impairment, with clinical significant depressive psychopathology being reported in around 31% of patients. Psychopathological features are the same observed in MD, along the same gradient of severity, and including a typically melancholic cognitive vulnerability. Neurocognitive impairment could possibly separate from depression in the long term, but not in the first year after infection, and it is largely overlapping with persistent cognitive deficits described in MD. We will discuss pathogenetic mechanisms shared by both, MD and post-COVID depression, with a specific emphasis on: (i) spread disruption of white matter microstructure, reduced grey matter volumes in anterior cingulate cortex, and abnormal functional connectivity in the cortico-limbic circuitries; (ii) abnormal cell trafficking across the blood brain barrier, essential for brain maintenance and repair in healthy conditions; (iii) altered immuno-inflammatory setpoints as observed in the peripheral blood, known to parallel white and grey matter abnormalities in the brain, and recently shown to disrupt neurovascular coupling and spontaneous neural activity. We suggest that post viral depression provides an invaluable model illness for the study of immune-inflammatory mechanisms involved in the pathogenesis of mood disorders, to identify new targets for treatment, with the aim of restoring mental health and brain homeostasis.

**Disclosure of Interest:**

None Declared

